# Book Reviews

**Published:** 1831-09

**Authors:** 


### BIBLIOGRAPHICAL NOTICES.

*Elements of Practical Midwifery; or, Companion to the Lying-in Room.* By Charles Waller, Consulting Accoucheur to the London and Southwark Midwifery Institution; Lecturer on Midwifery and Diseases of Women and Children. *Second Edition, with Additions.*—18mo. pp. 200; plates. Highley, London.

It was well said by the author of the "Rambler," that it is not without good effect that men of proper qualifications write in succession on the same subject, although much that is new remains not to be stated. The same ideas may be delivered more intelligibly by one than by another, or with attractions that may instruct minds of different forms and capacities. We fear not, therefore, the accumulation of elementary works, when the number is augmented by a writer like Mr. Waller, whose ample experience and ability enable him to avoid any practical errors that may have crept into the labours of his predecessors, and to state briefly and correctly the leading points of his subject, for the information of the juniors of the profession. This is just the kind of book we should be inclined to put into the hands of the obstetric student, before he entered upon the perusal of more elaborate treatises on the practice of midwifery: it is, in fact, a grammar of obstetric science, arranged with judgment, perspicuously written, and unincumbered (as all elementary works ought to be, and so seldom are,) with hypothetic speculations.

The principal additions to the present much-improved edition will be found in the sections on puerperal diseases, and the mode of introducing the female catheter.

---

*Advice to the Public for the Prevention and Cure of the Asiatic Cholera.* Translated from the German of Dr. J. R. Lichtenstadt, Professor of Medicine, &c.—8vo. pp. 20. Souter, London.

This little brochure effectually answers the purpose for which it is intended: it conveys in a concise, and very intelligent form for the non-professional reader, such a description of cholera as will enable him to ascertain the presence of the disease, together with some good general rules for its prevention and treatment, where the assistance of the physician cannot be obtained.

*Recherches sur la Traitement du Cancer, par la Compression Méthodique simple ou combinée, et sur l'Histoire génerale de la même Maladie: suivies de Notes, 1, Sur les Forces et la Dynametrie Vitáles; 2, Sur l'Inflammation et l'Etat Febrile.* Par J. C. A. Recamier, Medecin de l'Hotel Dieu de Paris, &c. Tomes I. and II.—Pp. 560 and 732; Planches.

Although every professional reader must allow that these volumes contain an immense body of important information, it is probable, we think, that many will feel some scruples in admitting that all the cases related by the talented author, under the denomination of cancer, were really of a carcinomatous character. But even if very strong doubts should be felt upon this point, it cannot but be conceded to M. Recamier, that the treatment he recommends is capable of relieving, and even sometimes of curing, cases of disease, which, if not strictly cancerous, would run their perhaps fatal course, in spite of all ordinary modes of practice. As it will be necessary to peruse the whole work with care and diligence, in order justly to appreciate the doctrines and rich practical illustrations with which it abounds, we shall confine ourselves to a very brief notice of it, intending only to attract the attention of our surgical brethren to its merits and utility.

After having laid down the plan of his work, the author says, that if the reader will follow the order in which the materials are presented, he will find himself associated in all the researches, and in the same situation he was in while collecting them. In this assurance, M. Recamier estimates too highly his powers of describing disease: we grant they are great, but still no verbal description of disease can be so satisfactory as the sight of it.

It appears that, in 1817, a remarkable instance of carcinomatous breast came under M. Recamier's notice. Internal remedies had failed, and such was the nature of the case that an operation was inadmissible. He tried compression with such good effects as to make a strong impression upon his mind. It was not, however, till 1825 that he entered into a course of practice and observation, from the collective results of which the work before us originates.

M. Recamier differs, and wisely too, from the prevailing custom of first advancing his theories and general principles: he commences with facts and data, and then proceeds to draw his conclusions. The first part contains the history of sixty-two cases of cancer of the breast; the second part comprises the history of thirteen cases of cancer in other parts of the body: all these cases are detailed very minutely, and with great apparent accuracy. M. R. adopts the following classification of cancerous diseases of the mammary glands:

"1. Some form diffuse engorgements in the parenchyma of these organs.

"2. Others constitute isolated tumors, which seem to involve the proper tissue of the breasts only consecutively.

"3. Others partake of the characters of parenchymatous engorgement and of isolated tumors.

"4. Others have, as their most prominent phenomenon, local neuralgic pains, which either precede or accompany the developement of the engorgements or the tumors.

"5. Others, with engorgements, tumors, and pains, present softenings (*ramollissemens*), or ulcers, more or less superficial or deep, but always possessing the refractory character of cancerous affections."

If a gentle, equal, and continued compression be applied to the first class of cases, diffuse engorgements; and the density of the tumor diminishes throughout its whole extent, and the breast return to its natural state, without atrophy, and without insulated and dense kernel remaining, entire success may be hoped for. If the engorgement should diminish, leaving still a distinct kernel, which increases in density, it takes on the condition of a circumscribed tumor: but if, instead of being resolved or becoming circumscribed, the engorgement attacks other parts of the breast, the treatment will fail, whatever external or internal remedies may be employed. The resolution of circumscribed tumors by compression is more difficult, because they are more dense and isolated: in some cases, however, it may be accomplished, but in others it is impossible. When the treatment is to succeed, the tumor gradually loses its insulated character, and its kernel daily becomes less dense. It is evident, then, that in circumscribed tumors, complete success is obtained by compression, by bringing them to the condition of diffused engorgement.

"3. *Simultaneous diffuse engorgements and circumscribed tumors of the breast.* In very fleshy persons, a circumscribed tumor in a mammary gland is often connected with a general affection of the organ and of the cellular tissue which surrounds it; and there is afterwards associated with it engorgement of the lymphatic ganglions, and even of the opposite mammary gland. If this sort of tumors be compressed, a part of the diffuse engorgement is dissipated, but the principal kernel resists, and obstinate infiltrations supervene in the neighbourhood; if an operation be performed, there is a relapse, whether compression be employed after the operation, or not until after some delay.

"4. *Tumors or engorgements accompanied with neuralgia.* In very irritable subjects, engorgements are sometimes developed, in which neuralgic pains form a principal trait in the disease, whether they precede or follow it. The author has seen neuralgia precede by several years, not only the cancerous engorgements of the breasts, but likewise those of other organs. Engorgements and tumors of this sort are very refractory.

"5. *Tumors with softening.* In whatever manner the disease may commence, there are cases where, when medical aid is first required, points of softening are found to exist. In this state, compression appears to be useful only for disgorging the circumference of the tumor, in order to proceed afterwards with more facility to extirpation, if we have not already to manage a case of relapse. Compression upon a softened kernel is never successful.

"6. *Ulcerated cancerous tumors.* In eight cases, Professor Recamier was not consulted, until after the ulceration of the cancerous tumors. Compression, in this case, did not seem to him applicable with advantage, before he had got rid of the ulcer, and his success was the more decisive, in proportion as the riddance was more complete."

For an account of the particular modes in which M. Recamier applies pressure in various cases, we must refer to the work.

*Cauterization.* The author employs the concentrated nitric acid, to each ounce of which he adds half a drachm or a drachm of crystallized nitrate of mercury. The part to be cauterized is placed in the most favorable situation for keeping the caustic applied to it, and a quantity of the nitrate is employed proportioned to the extent he intends to give to the eschars. The caustic liquor is applied by means of dossils of lint held in dressing forceps. If he wishes the caustic to act to a considerable depth, he allows the dossils to remain on the parts to be cauterized, pressing from time to time upon the lint with the end of the forceps, and watching at the same time the neighbouring skin, which he wipes immediately if the caustic should spread. Acute pain is produced by this treatment, and fever may supervene. When the cauterization is finished, a strong solution of opium may be applied to remove the pain. After cauterization, compression should be continued, if the patient can bear it.

*Excision.* The facility of removing the *whole* apparent disease by excision, and the impossibility of accomplishing this very important object in many cases by cauterization, appears at once to decide the surgeon in favor of the former: but relapses are more frequent after excision alone, than after cauterization. In five cases M. Recamier has employed compression and cauterization before resorting to excision, and in none of these cases has the disease returned.

*Internal treatment.* At Vienna, Storck and others have employed cicuta with, it is said, decided success: in France, this remedy has failed. M. Recamier has examined carefully the effects of cicuta, under different modes of managing the patient, and he has ascertained that they differ very much according to the quantity of aliment allowed. Cicuta, with a nutritious diet, produced but little effect upon the chronic engorgements; but the effect was evident when the ordinary quantity of food was greatly diminished. Hence he reflected maturely on the *cura famis* of Callissen, and he found that, if hemlock often fails, it is not from its want of power, but that, to obtain all its advantages, it is absolutely necessary to join its employment with a very severe regimen.

The following are the general results of M. Recamier's researches:

"1st. Of the one hundred patients who have come to him to be treated for cancerous affections, sixteen appeared to be entirely incurable. Of the remaining eighty-four, thirty have been completely cured by compression alone: twenty-one, upon whom compression was employed, experienced only an amelioration, but a very notable one; fifteen have had cancers extirpated, either by excision alone, or rather by excision combined with compression: six have had them removed by compression combined with cauterization: in the other twelve cases, the affection was absolutely unyielding.

"2d. Some tumors, similar, or at least analogous to those which degenerate into incurable cancers, got well by methodical compression and by some other external and internal means.

"3d. When compression has given to mammary engorgements which have not yet degenerated, a disposition towards resolution, this continues even after discontinuance of compression; but if the engorgement has degenerated, and if, after having obtained a great diminution, we leave off compressing the remaining hard and isolated kernel, we must expect the engorgement to regain its former volume, and its degeneration to proceed more rapidly.

"4th. Compression may assist in preventing relapses after excision.

"5th. The advantages derived from compression upon the remains of an ulcerated cancer in one of the author's cases, are not obtained in the same degree in some analogous cases.

"6th. Resolution of chronic mammitis is very much promoted by compression alone, or associated with local bleeding, &c.

"7th. Different uterine engorgements are resolved, by compressing the uterus by means of a pessary made in the form of a hollow cone, and pierced at its extremity.

"8th. The os tincæ and the uterus, having become cancerous, may be removed with safety, as long as the disease is circumscribed.

"9th. From these results, the author thinks himself allowed to conclude that, if compression be employed early, that is, before the degeneration of the engorgements which are susceptible of it, a great number of them may be resolved; and that the excision of cancers of the breast, so often followed by a relapse, when the tumors are not encysted, will become more and more rare.

"Of one hundred cases, two occurred in males and ninety-eight in females: fifty in the left breast; thirty-three in the right breast; three in both breasts; five in the uterus; three in the face; one in the tongue; one in the back; one in the rectum; one in the stomach.

"Of the sixty-two patients affected with cancer in the breast, whose histories are contained in Part I. sixteen had received blows on the part; thirty-nine had experienced no local violence; five have had, in their families, persons affected with it; two, who might be supposed to have inherited it, had received blows; twenty-one had axillary engorgements; four had subclavicular engorgements; and three had supra-clavicular engorgements."

---

*A Popular Treatise on the Teeth and Gums, and Diseases attendant on them. Designed for the Use of Families.* By JOHN WINCKWORTH.—4to. pp. 52; plates. Renshaw and Rush.

WE are by no means convinced that much benefit is often derived by the public from "Popular Treatises" on medical subjects. From such sources very superficial instruction is to be obtained; just enough, in fact, to make people believe they know something of that, of which, after all, they can know nothing. Such works may be of service to their authors, and perhaps there is no great harm in this species of advertisement. We make these observations generally, and without meaning them to apply particularly to Mr. W.'s work, which contains many useful hints for the general reader, and some which may be consulted with advantage by the surgeon, or professed dentist.

---

*The Art of Cupping: being a brief History of the Operation, from its Origin to the present Time; its Utility; Minute Rules for its Performance, &c.* By GEORGE FREDERICK KNOX, Cupper at the Westminster Hospital.—Highley, and Underwood, London.

SUFFICIENT opportunities of performing the operation of cupping cannot fall to the share of any practitioner, except, indeed, a professed cupper in large and populous towns, to enable him to acquire, by practice alone, that manual tact and dexterity which it so essentially requires. Some rules and precautions must be required by the unpractised pupil in the art of cupping, to enable him to satisfy himself or his patient, and in this little essay of Mr. Knox's every necessary instruction is contained, both as to the best modes of performing the operation in various parts of the body, and the proper selection of instruments.

---

*An Attempt to simplify the Treatment of Sexual Diseases.* By JAMES THORN, Member of the Royal College of Surgeons.—8vo. pp. 240. Highley, London.

THE compilation of this book may have cost the author some pains, but a less perfect or satisfactory digest we have never seen. We are at a loss to know for what class of readers it can be intended; surely not for surgeons, and as surely not for pupils; as, on the one hand, the preface truly declares that the author cannot even hope "to lay down any claims to originality (either) *in his views or mode of treatment*," and, on the other, although some comparatively unimportant points are dwelt on with tedious prolixity, many most interesting sexual diseases are omitted altogether. Indeed, the titlepage to this production, as Horace Walpole said of one of his own prefaces, is like the show-cloth to a sight: there is more entertainment promised on the outside than is contained within. Can it have been written for the public good? No, it is not fit for general perusal. In few subjects does true delicacy of mind shew itself with greater advantages than in the discussion of the diseases incidental to the sexual organs; all prurient ideas, all pandering to the passions, should be carefully banished. We certainly do not err when we opine that this volume was written by the author neither for the profession, pupils, nor the public, but for himself, and for himself alone.

